# New support system using a mobile device for diagnostic image display and treatment of acute stroke in Japanese depopulated areas

**DOI:** 10.1186/cc13644

**Published:** 2014-03-17

**Authors:** T Kageji, H Oka, F Obata, K Tani, H Bando, R Tabata, M Kohno

**Affiliations:** 1 Tokushima University Hospital, Tokushima, Japan; 2 Tokushima Prefectural Kaifu Hospital, Kaifu, Japan; 3 The University of Tokushima Graduate School, Tokushima, Japan

## Introduction

Stroke is the main cause of deterioration of activity of daily living in Japan. The social problem in Japan is the difference in medical quality between the urban and depopulated areas. To improve the problem, telemedicine using a mobile device between general physicians and stroke specialists became important with the increasing demand for rapid and correct diagnosis for treatment of acute stroke. We developed a system for rapidly exchanging diagnostic images and clinical information in depopulated areas to develop the standard thrombolytic therapy using alteplase for acute ischemic stroke [1,2].

## Methods

A system was consisted of communicating patient data and imaging between the hospital system and participating staff members in and out of the hospital using mobile devices. The system can transfer clinical data and large volumes of CT and MRI, and expert opinion in real time. We developed the system (k-support) in the Kaifu area, which is a typical depopulated area in Tokushima Prefecture, Japan, between the general physicians in Tokushima Prefectural Kaifu Hospital and specialists in stroke and cardiovascular disease at Tokushima University Hospital from February 2013.

## Results

The k-support system was managed in 102 emergency patients, 65 patients (64%) were classified as neurological disease, 41 (40%) as stroke, 11 (11%) as head injury, two (2%) as epilepsy. The detail of stroke was ischemia in 35 (34%), hemorrhage in four (4%) and SAH in two (2%). Two ischemic stroke patients were treated with intravenous thrombolysis of alteplase using the k-support system and a 'drip- and-ship' paradigm. One patient using alteplase showed complete recanalization of the middle cerebral artery. The consultations resulted in hospitalization in 42%, transfer in 37% and return home in 20%.

## Conclusion

Before introduction of the k-support system, the standard thrombolytic therapy using alteplase for acute ischemic stroke could not operate in the Kaifu area due to the absence of stroke specialists and the long distance to a neighboring stroke center. The telemedicine system using a mobile device as the k-support system can be used anytime, anywhere and by anyone. This system can communicate with the doctors, between general physicians in depopulated areas and specialists in urban areas, and may become a useful tool for acute patient management in not only stroke but also other emergency diseases.
